# The Impact of Frailty on Morbidity and Mortality following Open Emergent Colectomies

**DOI:** 10.1155/2017/5126452

**Published:** 2017-09-06

**Authors:** Dominick V. Congiusta, Prashanth Palvannan, Aziz M. Merchant

**Affiliations:** New Jersey Medical School, Department of Surgery, Rutgers Biomedical and Health Sciences, Newark, NJ, USA

## Abstract

**Background:**

Elderly and frail patients undergo open emergency colectomies and are at greater risk for complications. The relationship between frailty and open emergent colectomies is yet unexplored.

**Objective:**

The purpose of this study was to evaluate the relationship between frailty and outcomes after open emergent colorectal surgery.

**Design:**

Using the American College of Surgeons National Quality Improvement Program database, a validated modified frailty index was used, along with logistic regression, to assess the relationship between frailty and outcomes.

**Main Outcome Measures:**

Outcomes included mortality (primary), Clavien-Dindo Complication Grade >3, reintubation, ventilator >48 hours, and reoperation (secondary).

**Results:**

The rates for 30-day mortality, Clavien-Dindo Grade >3, reintubation, ventilator > 48 hours, and reoperation in our cohort were 16.6%, 36.9%, 8.6%, 23.9%, and 15.0%, respectively. There was a statistically significant increase in prevalence of all outcomes with increasing frailty.

**Limitations:**

A causal relationship between frailty and complications cannot be established in a retrospective analysis. Also, extrapolation of our data to reflect outcomes beyond 30 days must be done with caution.

**Conclusions:**

Frailty is a statistically significant predictor of mortality and morbidity after open emergent colectomies and can be used in an acute care setting.

## 1. Introduction

Nonelective open colectomies are among the highest national burden for acute care surgery, with significant morbidity, mortality, and hospital cost [1]. Indications for surgery include perforation, bleeding, and obstruction with a variety of underlying diagnoses such as diverticulitis, cancer, and colitis. While many acute care surgical procedures are done in younger, healthier populations, emergent open colectomies tend to be performed in elderly patients (average of 67 years) with comorbidities [2]. Moreover, emergent colectomies are associated with greater mortality and morbidity than those done electively [3]. In an effort to clarify this increased risk, studies have reported many factors, such as age, American Society of Anesthesiologists (ASA) Grade, and renal failure [4, 5]. However, the best predictor of surgical outcomes in this elderly cohort still remains elusive.

Currently, this population represents the fastest growing segment in the United States [6]. Almost fifty percent of all operations are performed on patients over the age of 60. As such, patient frailty is becoming increasingly recognized as an important predictor of surgical outcomes and a proxy for physiological reserve in the elderly [7]. Frailty has many definitions in the literature but generally refers to a decrease of physiological reserve giving rise to vulnerability separate from the normal aging process and thus correlates with morbidity. It can be described by two quantifiable models [7, 8]. The first is a physical-characteristic based model that incorporates unintentional weight loss, grip strength, exhaustion, walking speed, and physical activity [9]. The second is the Canadian Study of Health and Aging Frailty Index (CSHA-FI), which incorporates 70 clinical variables measuring medical, psychological, and functional capacity as an assessment of deficit accumulation [10].

Velanovich et al. derived a separate measure based on the CSHA-FI using 15 of the original 70 variables and termed it a “modified frailty index” (mFI) [11]. This measurement offers surgeons the ability to rapidly evaluate risk from a patient's medical history. Other models of frailty require variables that are not feasibly collected in emergency situations and delay care. As a function of simple history alone, the mFI can be readily transferable to any database that has information on patient history. Our research has shown that it has been shown to have strong predictive value in a variety of surgical subspecialties and procedures in NSQIP and other databases [7, 8, 12]. Due to its practicality and growing utility in the surgical field, we chose to investigate the mFI's use in colorectal surgery.

This model has not been used to assess outcomes in patients undergoing emergent open colectomies. Understanding the impact of frailty, via the mFI, can elucidate the specific risks associated with this procedure. In this manuscript, we demonstrate the use of the mFI as a predictor of morbidity and mortality in those undergoing nonelective open colectomy.

## 2. Methods

### 2.1. Data Source and Study Population

Institutional Review Board approval (exemption) was obtained. The American College of Surgeons National Quality Improvement Program (NSQIP) is a database that has collected deidentified data from voluntarily participating hospitals since 2005. NSQIP's data has been validated with rigorous quality control measures and through numerous studies.

We performed a retrospective analysis of the data in NSQIP from 2005 to 2012 and chose individuals who underwent open colectomies based on Current Procedural Terminology (CPT) Codes (Table 1). Cases were included if an open colectomy was listed in either the principle or any of the secondary CPT codes available in NSQIP. This allowed us to include those who might have been erroneously excluded if a colectomy was not listed as the primary procedure. It also allowed us to include any laparoscopic cases that may have been converted to laparotomies or other cases that required a colectomy. Next, we selected for only emergent procedures. Finally, patients missing any of the 15 variables needed to calculate mFI (Table 2) were excluded.

### 2.2. Modified Frailty Index (mFI)

The mFI was calculated by ascribing each variable with a value of 1, if positive, and 0, if negative. These values were then added together to determine each patient's mFI score. Thus, the mFI score represents a sum total of health deficits. Patients were grouped into one of five cohorts, from 0 through ≥4. As patients with an mFI score of ≥4 constituted only 13% of our population, they were grouped together to provide comparable cohort sizes for analysis. Previous data have demonstrated validity for the mFI for as few as 10 variables [13, 14]. However, we chose the most commonly utilized mFI, which includes 15 NSQIP variables [8]. As described by Velanovich et al., some variables should be combined due to either similar pathophysiology or risk factors; this prevents accounting for the same problems twice. Thus, the 15 NSQIP variables are sorted into 11 “mFI variables,” which are then added together to form the mFI score (Table 2).

### 2.3. Outcomes of Interest

Our primary outcome of interest was 30-day mortality. Secondary outcomes were Clavien-Dindo Grade ≥ 3, reintubation, return to the operating room, and dependence on a ventilator after 48 hours postoperatively. The Clavien-Dindo Classification system is a ranking tool used to sort postoperative complications into categories based on the magnitude of interventions required for management [15]. Grades 3 and greater indicate severe complications that require more extensive treatment measures and have been used as a cutoff point in several studies to indicate high severity [16, 17]. They include reoperations, life-threatening complications requiring intensive care, and complications leading to death. Complications were categorized based on their typical management (Table 3).

### 2.4. Predictor Variables

In addition to calculating the mFI score, the NSQIP database was queried for information on age, American Society of Anesthesiologists (ASA) classification, gender, prior operation within 30 days, wound infection, disseminated cancer, renal failure, dyspnea, smoking status, and ventilator dependence 48 hours prior to surgery. We analyzed the relationship between the mFI score and these variables with our outcomes.

### 2.5. Statistical Analysis

Chi-square analysis was conducted between the predictor variables (age, ASA Class, gender, prior operation within 30 days, wound infection, disseminated cancer, renal failure, dyspnea, smoking status, and ventilator dependence 48 hours prior to surgery), mFI score, and each outcome variable. Multivariate logistic regression was then used to compare the impact of mFI on each of the outcomes, adjusting for the predictor variables. Results were reported as odds ratio (OR) with 95% confidence interval for both primary and secondary outcomes (Tables 7 and 8, resp.). In order to specifically assess the use of mFI in the elderly, another set of multivariate logistic regression models was conducted for an elderly cohort, defined as age ≥ 60 years (Tables 8 and 9). In all models, independent variable odds ratios were determined by controlling for remaining causal or closely correlated causal variables. Data were entered and analyzed using Statistical Package for the Social Science (SPSS) (International Business Machines, Corp., Armonk, NY).

## 3. Results

### 3.1. Demographics

Of the 148,637 open colectomies in NSQIP from 2005 to 2012, 19,649 emergent open colectomy cases were included in the final analysis after exclusion criteria were applied. Demographics and preoperative comorbidities were recorded (Table 4). Of note, almost 63% of patients in the cohort were over the age of 60, and almost a fifth were octogenarians or older. Moreover, of those classified with an mFI score of 0, 22 percent were younger than 41 years. Conversely, mFI cohorts of 3 and 4 or greater had a predominance of older patients with 81.7% and 84.2% being at least 60 years of age. These two groups represented a quarter of the study population (Table 5).

The study population had an overall mortality rate of 16.6% (Table 6). The most common independent variable associated with patients who died was ASA Grade 4 or more. 72% (2,005) of 3,264 total deaths were in patients under this particular classification. This represented 31.5% of all patients with an ASA score of at least 4, almost double the overall study population's mortality rate.

### 3.2. Univariate Analysis of Comparison Predictor Variables and mFI

Chi-square analysis of various predictor variables with 30-day mortality revealed the following were all significantly associated: age, ASA Grade, disseminated cancer, dyspnea, gender, prior operations, procedure with anastomoses, race, renal failure, smoking, systemic sepsis, ventilator dependence > 48 hours prior to surgery, and wound infection. Subsequent univariate analysis of these thirteen variables and mFI score revealed a significant correlation in the following: age, disseminated cancer, dyspnea, prior operations, smoking, ventilator dependence for >48 hours prior to surgery, and wound infection. Of these seven, age, dyspnea, and ventilator need had the strongest association with mFI (*X*^2^_age_ = 3,907, df = 12; *X*^2^_dyspnea_ = 2,369, df = 4; *X*^2^_ventilator_ = 2,112, df = 4). Effect size calculation showed that only ventilator use and dyspnea had a moderate effect on mFI score, while age and the remaining variables had minimal effect (Cramer's *V*_dyspnea_ = 0.35; Cramer's *V*_ventilator_ = 0.33).

### 3.3. 30-Day Mortality

**Figure 1:**
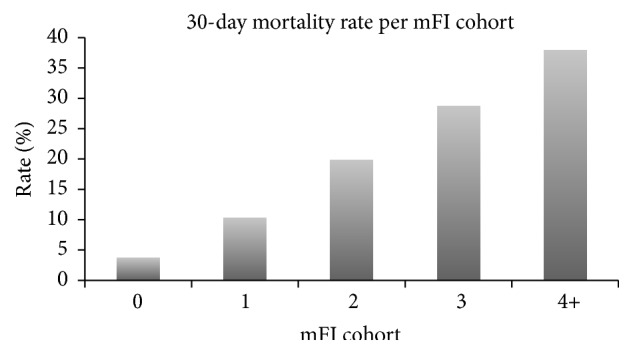
30-day mortality rate for each mFI cohort (*p* < 0.05). mFI = modified frailty index score.

Overall mortality rate was 16.6%. Univariate analysis revealed that of those who had mFI scores of 0, 1, 2, 3, and ≥4, 30-day mortality rate was 3.8%, 10.3%, 19.8%, 28.7%, and 38.0%, respectively (*p* < 0.05; Figure 1). Logistic regression revealed that the odds of 30-day mortality increased significantly with each increase in mFI score after adjusting for comorbid conditions (Table 7).

### 3.4. Secondary Outcomes

**Figure 2:**
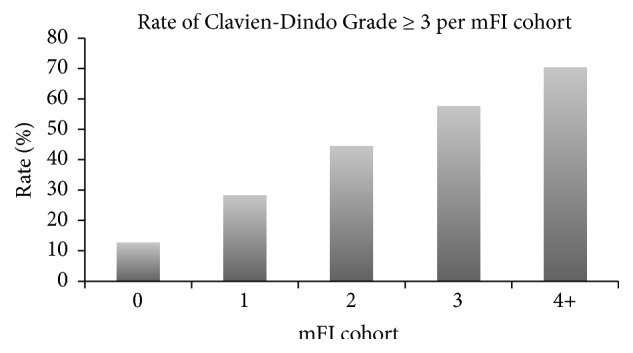
Clavien-Dindo Grade ≥ 3 complication rate for each mFI cohort (*p* < 0.05). mFI = modified frailty index score.

**Figure 3:**
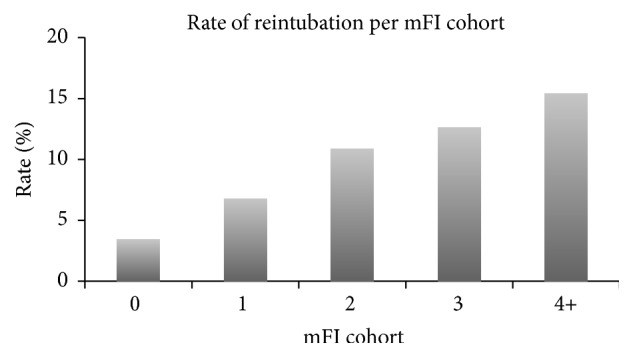
Reintubation rate for each mFI cohort (*p* < 0.05). mFI = modified frailty index score.

**Figure 4:**
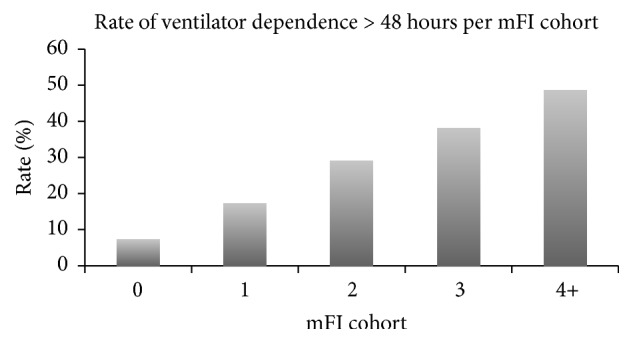
Rate of dependence on ventilator > 48 hours for each mFI cohort (*p* < 0.05). mFI = modified frailty index score.

**Figure 5:**
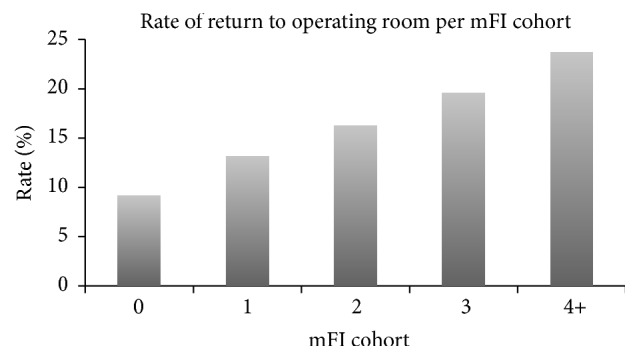
Rate of return to operating room for each mFI cohort (*p* < 0.05). mFI = modified frailty index score.

The overall rates of Clavien-Dindo Grade ≥ 3 complications, reintubation, dependence on ventilator, and return to operating room were 36.9%, 8.6%, 23.9%, and 15.0%, respectively (Figures 2–5). As expected, logistic regression revealed that the odds of having each of the secondary outcomes increased with increasing mFI score, after adjusting for comorbid conditions (*p* < 0.05) (Table 8).

### 3.5. Impact of mFI on Primary and Secondary Outcomes in Patient ≥ 60 Years

Logistic regression revealed that the odds of 30-day mortality in patients aged 60 years and older increased significantly with concurrently increasing mFI score. Importantly, patients with mFI score 2, 3, or ≥4 had significantly increased mortality than those with score of 0. The mFI 3 and ≥4 cohorts had a 41% and 46% increase in 30-day mortality, respectively. Aside from mFI, disseminated cancer, age > 80, and systemic sepsis were the three strongest predictor variables (Table 9).

Similarly, models for ventilator dependence for >48 hours prior to surgery, reintubation, return to OR, and Clavien-Dindo Grade ≥ 3 demonstrated the significant predictive value of increasing mFI score on these secondary outcomes. In all these cases, mFI scores of 3 and ≥4 were associated with significantly increased odds. History of smoking, dyspnea, ventilator dependence for >48 hrs prior to surgery, procedure with anastomosis, and systemic sepsis were also significant for all four secondary outcomes of interest (Table 10).

## 4. Discussion 

Our study reveals that frailty is an important predictor of mortality in patients undergoing nonelective colectomies. The overall 30-day mortality of almost 17% within our NSQIP cohort underscores the perils of this important treatment for colorectal emergencies. In addition, the overall complication rates for Clavien-Dindo Grade ≥ 3, reintubation, ventilator dependence ≥ 48 hours, and return to the operating room were 36.9%, 8.6%, 23.9, and 15.0%, respectively, indicating the high perioperative morbidity within this acute care surgical population. Moreover, increasing patient frailty was significantly associated with an increasing mortality rate. Indeed, in our study, frailty was among the strongest predictors for both the primary and secondary outcomes.

Our findings of increased morbidity associated with open colectomies has been consistent documented in current literature. For example, one study showed that those who underwent open colectomy had high rates of anastomotic leak (3.4%), hematuria (6.8%), urinary tract infections (4.5%), and ureteral injury (2.2%); mean hospital charges were also high ($14,863) [18]. Another recent study showed that morbidity in open colectomies for colorectal cancer in patients over 80 years was 52.7%; mortality was 16.5% [19]. Emergent colectomies represent an extremely important subgroup within acute care surgery, which warrants close analysis. It is well known that emergency colectomies are associated with significant morbidity and mortality in the elderly, sometimes as high as 26% depending upon the indication and type of colectomy [18, 20–23]. In fact, when looking at overall complex intestinal resection in the elderly patient, the greatest predictor of mortality was the emergent status of an operation [24].

With the current aging population, identifying frail patients is an essential component of the preoperative risk assessment, one that may curtail the rising postoperative complications and mortality. Having data, such as these and ours, can inform crucial conversations with patients and families in these extremely stressful acute care surgical scenarios. Within the NSQIP database, emergency colectomies are common and constitute a large proportion of emergent cases (9.5%). Resection of the colon has the highest burden rank of emergency general surgery procedures for complications and costs, indicating the impact that this procedure has on the current healthcare system [1].

The mFI is both reliable and feasible to use in emergency colorectal surgery with distinct clinical advantages over alternative models. Faynsod et al. used the Nationwide Inpatient Sample (NIS) to develop and internally validate a risk predictor model for emergency colectomy [21]. In their large study of approximately 300,000 patients, a logistic regression model identified 8 variables for the predictor model. Furthermore, using the same NIS, Masoomi et al., evaluated 900,000 patients who underwent elective and emergent colectomy to identify variables that were predictive of in-hospital mortality after colectomy [25]. Many of these variables identified in both studies also appear in the 15-variable mFI used in our analysis. Our study is therefore congruent and in agreement with some of the large administrative database studies on emergency colon surgery.

While such studies have sought to identify predictors using large national datasets, ours specifically addresses the relationship between frailty and outcomes, by utilizing a previously developed and validated scoring index (Velanovich et al.). The mFI includes important factors in elderly patients, such as impaired sensorium (dementia), functional status (disability), and a history of stroke. These latter components of elderly physiologic reserve have been independently verified to pose increased odds of postoperative mortality in elderly surgical cohorts and distinguish the mFI from other comorbid indexes [26, 27]. Moreover, frailty is an important metric that is being increasingly used to predict postoperative outcomes in both our aging population [1–3, 6, 8] and emergency surgery [28, 29].

One of the inherent problems in emergency surgery is time restriction, which prevents thorough testing, comprehensive risk assessment, and possible medical optimization of management. These compound and produce stressful situations with high complication rates. As discussed by Kermani et al., existing risk calculators for colectomies include data that cannot be feasibly collected at the bedside [20]. Such models include the NSQIP Risk Calculator, APACHE, and Charlson Comorbidity Score. They use complex formulas or require lab values and clinical data that might delay critical decisions in emergency situations [30]. A reliable and rapid form of assessment is needed in such circumstances. The mFI addresses these issues through its practicable calculation of perioperative risk for frail patients by using medical history variables. Thus, it is similar to existing comorbidity indexes but has particular advantages. It allows surgeons to rapidly ascribe a more consistent risk score to patients, whereas physiologic variables used in other models can change quickly and make this difficult in both elective and emergency settings [20]. Its growing popularity among the surgical research community is evidence of its potential utility.

Our results support the predictive value of the modified frailty index (mFI) in evaluating certain surgical morbidity as well, in addition to overall mortality. A statistically significant increase in 30-day mortality, Clavien-Dindo Grade ≥ 3, ventilator dependence 48 hours after surgery, return to the operating room within 30 days, and reintubation was found with each mFI cohort, particularly with mFI 3 and ≥4 in the general population.

We found that the mFI was also predictive in elderly patients alone. There was a statistically significant increase in 30-day mortality for patients with mFI of at least 2. Elderly patients with an mFI score of 2 or 3 had 47% and 72% increased odds of death within 30 days, and those with scores of ≥4 had twice the odds of death. When compared with the other predictive variables, mFI scores were one of the strongest predictors for mortality, and this trend was present with the other secondary outcomes as well. In the cases of postoperative ventilator dependence, reintubation, and Clavien-Dindo Grade of ≥3, mFI scores of 3 and 4 predicted at least 50% increased odds of morbidity. These data support mFI as a strong and consistent predictor of postoperative outcomes in the elderly population. Importantly, it effectively assesses preoperative risks in populations with an increase baseline of underlying comorbidities.

We used the topmost level of the frail cohort as mFI ≥ 4, realizing that this resulted in only 13% of the population as being extremely frail. To have a lower top level would have resulted in having patients that were not truly as frail as one would expect, especially since the model is based on deficits that increased and accumulate over time. To set the top level higher towards five, six, or more items in the mFI would have resulted in a quickly diminishing study population because we found that a relatively high number of patients who underwent emergency colectomy were missing multiple variables needed for the mFI.

Our analysis is limited due to its retrospective nature and because NSQIP only contains data up to 30 days after surgery. Therefore, extrapolation of this data beyond 30 days must be done with caution. We also excluded 90,492 patients due to lack of mFI data, which might have altered our results, but this was a necessary limitation in order to truly assess the validity of the mFI; we could not assume missing data meant a negative finding. Although NSQIP is a national database with wide reaching penetration, participation is still voluntary. Hence, our conclusions may not be wholly applicable for every hospital or provider, especially those that may have chosen not to participate in NSQIP. Despite these limitations, NSQIP demographic distributions tend to be representative of the United States population, and the data is collected accurately via a reproducible, validated collections methodology. A number of investigators have proven the validity and accuracy of NSQIP [31, 32].

Finally, while our study showed the impact of mFI in identifying high risk patients, it does not necessarily offer treatment options targeting potentially actionable comorbidities. Many of the factors involved in calculating mFI involve chronic medical conditions, such as a history of diabetes mellitus, hypertension, and myocardial infarction. These are often not addressable in acute care settings, preventing clinicians from reducing preoperative risk and altering postoperative morbidity and mortality. Using a validated and effective predictor index, like the mFI, can allow clinicians to recognize the impact of these measures. Although many other risk indices exist, many of them require long calculations that are not easily performed at the bedside. The mFI calculation can be done quickly through readily available elements in the history and the information is readily obtainable from patients or their nearest family members. When applied preoperatively, it can help inform patients and their families of their likely postoperative outcomes, it may aid physicians and families in informed decision making, and it may also help in allocating greater hospital resources to these patients from the onset. In addition, a related limitation is that some may construe the mFI index as a form of a multimorbidity index with additional terms, dependence, and impaired sensorium. This is a valid criticism of the mFI framework, and further work is needed to understand how the latter two terms contribute to the validity of the overall index.

This study analyzed frailty in all open emergent colectomies. The next step is to explore the mFI's role in common indications for the surgery, such as ulcerative colitis, cancer, and diverticulitis. In addition, further research analyzing the validity of the mFI in prospective settings will be critical in assessing its role in acute care surgery. Integrating this measure along with others, such as those that predict disease severity and treatment, may be useful in refining treatment guidelines in the near future.

## 5. Conclusions

The findings of our study demonstrate that the mFI is predictive of 30-day mortality, Clavien-Dindo Grade ≥ 3, dependence on ventilator 48 hours after surgery, return to the operating room within 30 days, and reintubation. We show that, consistent with existing literature, frailty is an important indicator for poor outcomes after surgery. These findings can help inform conversations with patients and families about risks of emergency surgery in this population. Identifying and classifying frail patients in this way allow for appropriate preoperative counseling and postoperative planning to potentially improve outcomes.

## Figures and Tables

**Table 1:** Open colectomy CPT Code descriptions.

CPT^a^ code	Description
44140	Colectomy, partial: with anastomosis
44141	Colectomy, partial: with skin level cecostomy or colostomy
44143	Colectomy, partial: with end colostomy and closure of distal segment (Hartmann type procedure)
44144	Colectomy, partial: with resection, colostomy or ileostomy, and creation of mucofistula
44145	Colectomy, partial: with coloproctostomy (low pelvic anastomosis)
44146	Colectomy, partial: with coloproctostomy (low pelvic anastomosis), with colostomy
44147	Colectomy, partial: abdominal and transanal approach
44150	Colectomy, total: abdominal without proctectomy, with ileostomy or ileoproctostomy
44151	Colectomy, total: abdominal, without proctectomy; with continent ileostomy
44155	Colectomy, total: abdominal, with proctectomy, with ileostomy
44156	Colectomy, total, abdominal, with proctectomy, with continent ileostomy
44157	Colectomy, total: abdominal, without proctectomy, with ileoanal anastomosis, including loop ileostomy, and rectal mucosectomy, when performed
44158	Colectomy, total: abdominal, without proctectomy; with ileoanal anastomosis, creation of ileal reservoir (S or J), including loop ileostomy, and rectal mucosectomy, when performed
44160	Colectomy, partial: with removal of terminal ileum with ileocolostomy

^a^Current Procedural Terminology.

**Table 2:** NSQIP^a^ variables to calculate mFI^b^.

COPD^c^ or recent pneumonia
Myocardial infarction
Congestive heart failure
Angina, previous coronary intervention, or previous coronary surgery
Diabetes mellitus
Transient ischemic attack or cerebrovascular accident
Cerebrovascular accident with neurological deficit
Hypertension requiring medication
Functional status (totally or partially dependent)
Impaired sensorium
Peripheral vascular disease or ischemic rest pain

^a^National Surgical Quality Improvement Program; ^b^modified frailty index; ^c^chronic obstructive pulmonary disease.

**Table 3:** Clavien-Dindo Complication Grade definitions.

Grade	Definition and variables
1	Postoperative complications not requiring intervention
	*Peripheral nerve injury, neurological deficit, renal insufficiency, superficial SSI* ^*a*^ *, or acute renal failure *

2	Postoperative complications requiring pharmacologic interventions
	*Deep SSI, organ space SSI* ^*a*^ *, sepsis, urinary tract infection, transfusion, pulmonary embolism, deep vein thrombosis, wound disruption or dehiscence, or pneumonia *

3	Postoperative complications requiring surgical, radiologic, or endoscopic interventions
	*Return to operating room*

4	Life-threatening postoperative complications requiring management in intensive care unit
	*Requiring ventilator > 48 hours, reintubation, cardiac arrest, progressive renal failure, myocardial infarction, or septic shock *

5	Postoperative complications leading to death
	*30-day mortality *

^a^Surgical site infection.

**Table 4:** Descriptive statistics.

Age	
≤40	1601 (8.1%)
41–60	5617 (28.6%)
61–80	8772 (44.6%)
≥81	3659 (18.6%)
Gender	
Male	9074 (46.2%)
Female	10529 (53.6%)
Race	
White	14216 (72.3%)
Black	2033 (10.3%)
Hispanic	968 (4.9%)
Other/unknown	1903 (9.7%)
Prior operation in past 30 days	1881 (9.6%)
Wound infection	1409 (7.2%)
Dyspnea	3854 (19.6%)
Smoking	4328 (22%)
Ventilator 48 hours prior to surgery	2118 (10.8%)
Renal failure	1032 (5.3%)
Disseminated cancer	1095 (5.6%)
ASA class^a^	
1	502 (2.6%)
2	3999 (20.4%)
3	7881 (40.1%)
4	6357 (32.4%)
mFI^b^	
0	5261 (26.8%)
1	5124 (26.1%)
2	4022 (20.5%)
3	2697 (13.7%)
≥4	2545 (13.0%)

^a^American Society of Anesthesiologists Class. ^b^Modified frailty index score.

**Table 5:** mFI cohorts by age.

Age (yrs)	mFI score				
	0	1	2	3	≥4
≤40 (8%)	1164	287	90	41	19
41–60 (29%)	2358	1528	897	452	382
61–80 (44%)	1427	2352	2037	1479	1478
≥81 (19%)	312	957	998	726	666

**Table 6:** 30-day outcomes.

Outcome	Total (*n* = 19,649)
Mortality	3264 (16.6%)
Ventilator > 48 hours	4704 (23.9%)
Reintubation	1694 (8.6%)
Return to operating room	2941 (15%)
Clavien-Dindo Grade	
0	8747 (44.5%)
1	843 (4.3%)
2	2803 (14.3%)
≥3	7256 (36.9%)
Superficial SSI^a^	1556 (7.9%)
Deep SSI^a^	463 (2.4%)
Organ space SSI^a^	1212 (6.2%)
Wound disruption	759 (3.9%)
Pulmonary embolism	265 (1.3%)
Renal insufficiency	353 (1.8%)
Acute renal failure	734 (3.7%)
Urinary tract infection	963 (4.9%)
Peripheral nerve injury	16 (0.1%)
Bleeding requiring transfusion	2111 (10.7%)
Deep vein thrombosis	536 (2.7%)
Sepsis	1653 (8.4%)
Septic shock	2174 (11.1%)

^a^Surgical site infection.

**Table 7:** Odds ratios of mortality predictors with 95% confidence intervals.

Variable	Death
Age > 80	6.02 (4.22–8.59)^*∗*^
Race (Black)	0.85 (0.72–0.99)^*∗*^
Female	1.05 (0.95–1.15)
On ventilator^a^	2.05 (1.79–2.34)
mFI^b^ = 1	1.32 (1.09–1.6)^*∗*^
mFI = 2	1.81 (1.49–2.2)^*∗*^
mFI = 3	2.11 (1.72–2.59)^*∗*^
mFI = 4+	2.41 (1.95–2.97)^*∗*^
Prior operation^c^	0.96 (0.83–1.11)^*∗*^
Wound infection	1.23 (1.05–1.44)^*∗*^
Disseminated cancer	3.12 (2.63–3.69)^*∗*^
Renal failure	1.42 (1.2–1.69)^*∗*^
Dyspnea	1.43 (1.29–1.59)^*∗*^
Smoke	1.19 (1.06–1.35)^*∗*^
Procedure with anastomosis	1.04 (0.93–1.16)
Systemic sepsis	1.66 (1.48–1.85)^*∗*^
ASA^d^ = 2	1.02 (0.37–2.86)
ASA = 3	3.12 (1.14–8.5)^*∗*^
ASA = 4	8.73 (3.2–23.84)^*∗*^

*∗* indicates significance at *p* < 0.05. ^a^On ventilator within 48 hours prior to surgery. ^b^Modified frailty index score (reference group mFI = 0). ^c^Prior operation within 30 days. ^d^American Society of Anesthesiologists Class (reference group ASA Grade 1). Results shown are logistic regression analysis, adjusting for predictor variables.

**Table 8:** Odds ratios of secondary outcome predictors with 95% confidence intervals.

Variable	Clavien-Dindo ≥ 3	Ventilator > 48 hours^a^	Reintubation	Return to OR^b^
Age > 80	1.95 (1.59–2.39)^*∗*^	0.97 (0.78–1.22)	1.33 (0.97–1.83)	0.47 (0.38–0.58)^*∗*^
Race (Black)	1.01 (0.89–1.14)	1.19 (1.04–1.35)^*∗*^	1.08 (0.91–1.29)	1.23 (1.08–1.41)^*∗*^
Female	1.02 (0.94–1.1)	0.95 (0.88–1.03)	0.94 (0.84–1.05)	0.88 (0.8–0.96)^*∗*^
mFI^c^ = 1	1.41 (1.25–1.59)^*∗*^	1.52 (1.31–1.76)^*∗*^	1.35 (1.1–1.66)^*∗*^	1.21 (1.05–1.4)^*∗*^
mFI^c^ = 2	1.83 (1.61–2.08)^*∗*^	2.02 (1.73–2.35)^*∗*^	1.79 (1.45–2.21)^*∗*^	1.32 (1.13–1.54)^*∗*^
mFI^c^ = 3	2.1 (1.82–2.43)^*∗*^	2.15 (1.82–2.54)^*∗*^	1.89 (1.51–2.37)^*∗*^	1.42 (1.20–1.69)^*∗*^
mFI^c^ = 4+	2.38 (2.03–2.78)^*∗*^	2.3 (1.93–2.73)^*∗*^	2.2 (1.74–2.78)^*∗*^	1.41 (1.18–1.69)^*∗*^
Prior operation^d^	1.15 (1.01–1.3)^*∗*^	1.21 (1.07–1.37)^*∗*^	0.94 (0.79–1.11)	1.42 (1.25–1.62)^*∗*^
Wound infection	1.34 (1.16–1.55)^*∗*^	1.37 (1.19–1.58)^*∗*^	1.15 (0.96–1.39)	1.14 (0.98–1.33)
Disseminated cancer	1.57 (1.35–1.83)^*∗*^	0.95 (0.8–1.12)	0.97 (0.76–1.23)	0.76 (0.62–0.94)^*∗*^
Renal failure	2.24 (1.85–2.7)^*∗*^	1.44 (1.23–1.7)^*∗*^	1.1 (0.89–1.36)	1.19 (1–1.42)
Dyspnea	1.54 (1.4–1.69)^*∗*^	1.36 (1.24–1.5)^*∗*^	1.43 (1.26–1.62)^*∗*^	1.21 (1.08–1.34)^*∗*^
Smoke	1.24 (1.13–1.37)^*∗*^	1.22 (1.11–1.35)^*∗*^	1.25 (1.09–1.42)^*∗*^	1.31 (1.18–1.45)^*∗*^
On ventilator	4.38 (3.71–5.17)^*∗*^	2.51 (2.2–2.85)^*∗*^	0.98 (0.82–1.17)	1.53 (1.33–1.75)^*∗*^
Procedure with anastomosis	0.86 (0.79–0.93)^*∗*^	0.75 (0.69–0.83)^*∗*^	0.95 (0.84–1.08)	1.29 (1.17–1.42)^*∗*^
Systemic sepsis	1.9 (1.75–2.06)^*∗*^	2.19 (1.99–2.42)^*∗*^	1.33 (1.17–1.51)^*∗*^	1.29 (1.16–1.42)^*∗*^
ASA^e^ = 2	2.55 (1.34–4.86)^*∗*^	3.11 (1.14–8.49)^*∗*^	9.14 (1.27–65.91)^*∗*^	2.09 (1.24–3.51)^*∗*^
ASA^e^ = 3	6.77 (3.57–12.84)^*∗*^	10.73 (3.97–29.03)^*∗*^	21.89 (3.05–157.13)^*∗*^	3.95 (2.35–6.61)^*∗*^
ASA^e^ = 4	19.13 (10.07–36.35)^*∗*^	27.06 (9.99–73.28)^*∗*^	26.22 (3.64–188.69)^*∗*^	5.99 (3.55–10.09)^*∗*^

*∗* indicates significance at *p* < 0.05. ^a^On ventilator within 48 hours prior to surgery. ^b^Operating room. ^c^Modified frailty index score (reference group mFI = 0). ^d^Prior operation within 30 days. ^e^American Society of Anesthesiologists Class (reference group ASA Grade 1). Results shown are logistic regression analysis, adjusting for predictor variables.

**Table 9:** Odds ratios of mortality predictors in patients ≥ 60 years with 95% confidence intervals.

Variable	Death
Age > 80	2.34 (2.22–2. 45)^*∗*^
Race (Black)	0.78 (0.60–0.97)^*∗*^
Female	1.01 (0.91–1.12)
On ventilator^a^	1.89 (1.73–2.04)^*∗*^
mFI^b^ = 1	1.18 (0.94–1.42)
mFI = 2	1.70 (1.47–1.93)^*∗*^
mFI = 3	1.98 (1.74–2.22)^*∗*^
mFI = 4+	2.37 (2.13–2.62)^*∗*^
Prior operation^c^	0.98 (0.87–1.11)
Wound infection	1.27 (1.10–1.44)^*∗*^
Disseminated cancer	3.00 (2.81–3.20)^*∗*^
Renal failure	1.27 (1.10–1.44)^*∗*^
Dyspnea	1.41 (1.29–1.53)^*∗*^
Smoke	1.24 (1.10–1.44)^*∗*^
Procedure with anastomosis	0.99 (0.87–1.11)
Systemic sepsis	1.71 (1.59–1.83)^*∗*^
ASA^d^ = 2	1.02 (0.37–2.86)
ASA = 3	1.02 (0.68–1.36)
ASA = 4	2.67 (0.94–4.40)

*∗* indicates significance at p < 0.05. ^a^On ventilator within 48 hours prior to surgery. ^b^Modified frailty index score (reference group mFI = 0). ^c^Prior operation within 30 days. ^d^American Society of Anesthesiologists Class (reference group ASA Grade 1). Results shown are logistic regression analysis, adjusting for predictor variables.

**Table 10:** Odds ratios of secondary outcome predictors in patients ≥ 60 years with 95% confidence intervals.

Variable	Clavien-Dindo ≥ 3	Ventilator > 48 hours^a^	Reintubation	Return to OR^b^
Age > 80	1.45 (1.16–1.74)^*∗*^	0.99 (0.88–1.10)	1.08 (0.94–1.22)	0.69 (0.55–0.82)
Race (Black)	0.89 (0.69–1.08)	1.19 (1.03–1.35)^*∗*^	1.13 (0.91–1.33)	1.35 (1.17–1.52)^*∗*^
Female	0.93 (0.81–1.05)	0.95 (0.85–1.05)	0.95 (0.85–1.05)	0.88 (0.76–1.00)
mFI^c^ = 1	1.30 (1.10–1.50)^*∗*^	1.29 (1.08–1.50)^*∗*^	1.07 (1.81–1.33)^*∗*^	1.04 (0.83–1.25)
mFI^c^ = 2	1.47 (1.27–1.67)^*∗*^	1.72 (1.52–1.92)^*∗*^	1.47 (1.21–1.73)^*∗*^	1.17 (0.96–1.39)
mFI^c^ = 3	1.64 (1.42–1.86)^*∗*^	2.06 (1.84–2.28)^*∗*^	1.56 (1.28–1.83)^*∗*^	1.28 (1.05–1.51)^*∗*^
mFI^c^ = 4+	1.96 (1.71–2.20)^*∗*^	2.44 (2.29–2.59)^*∗*^	1.90 (1.62–2.18)^*∗*^	1.35 (1.22–1.59)^*∗*^
Prior operation^d^	1.08 (0.87–1.29)	1.26 (1.11–1.41)^*∗*^	0.99 (0.79–1.19)^*∗*^	1.35 (1.19–1.51)^*∗*^
Wound infection	1.23 (0.98–1.47)	1.32 (1.15–1.49)^*∗*^	1.17 (0.95–1.38)^*∗*^	1.06 (0.87–1.25)
Disseminated cancer	1.34 (1.10–1.58)^*∗*^	0.94 (0.74–1.15)	0.97 (0.69–1.24)	0.68 (0.41–0.94)^*∗*^
Renal failure	1.45 (1.16–1.74)^*∗*^	1.44 (1.25–1.63)^*∗*^	1.06 (0.80–1.31)^*∗*^	1.15 (0.93–1.36)
Dyspnea	1.37 (1.22–1.52)^*∗*^	1.35 (1.24–1.45)^*∗*^	1.41 (1.27–1.55)^*∗*^	1.17 (1.04–1.30)^*∗*^
Smoke	1.47 (1.30–1.64)^*∗*^	1.35 (1.23–1.38)^*∗*^	1.29 (1.13–1.38)^*∗*^	1.45 (1.31–1.58)^*∗*^
On ventilator	4.69 (4.36–5.02)^*∗*^	2.44 (2.29–2.59)^*∗*^	0.88 (0.67–1.09)^*∗*^	1.52 (1.35–1.69)^*∗*^
Procedure with anastomosis	0.84 (0.72–0.96)^*∗*^	0.71 (0.60–0.82)^*∗*^	0.96 (0.82–1.10)^*∗*^	1.30 (1.18–1.42)^*∗*^
Systemic sepsis	1.65 (1.53–1.77)^*∗*^	2.20 (2.10–2.30)^*∗*^	1.28 (1.13–1.42)^*∗*^	1.34 (1.22–1.46)^*∗*^
ASA^e^ = 2	1.03 (0.68–1.38)	1.13 (0.83–1.43)	1.84 (0.87–2.81)	0.05 (0.01–0.09)
ASA^e^ = 3	1.76 (0.63–2.90)	2.94 (0.45–5.43)	3.84 (1.85–5.83)	1.97 (0.97–2.97)
ASA^e^ = 4	3.50 (2.37–4.64)^*∗*^	6.89 (5.46–8.32)^*∗*^	4.45 (2.46–6.44)	2.73 (1.55–3.91)

*∗* indicates significance at *p* < 0.05. ^a^On ventilator within 48 hours prior to surgery. ^b^Operating room. ^c^Modified frailty index score (reference group mFI = 0). ^d^Prior operation within 30 days. ^e^American Society of Anesthesiologists Class (reference group ASA Grade 1). Results shown are logistic regression analysis, adjusting for predictor variables.
